# Barriers to and Facilitators of the Implementation of the Central Chronic Medicines Dispensing and Distribution Programme in South Africa: A Scoping Review

**DOI:** 10.3390/pharmacy14070143

**Published:** 2026-09-29

**Authors:** Kalaba Nkonde, Nontobeko P. Mncwangi, Rubina Shaikh, Neelaveni Padayachee

**Affiliations:** 1 Department of Pharmacy and Pharmacology, Faculty of Health Sciences, University of the Witwatersrand, 7 York Road, Parktown, Johannesburg 2193, South Africa; rubina.shaikh@wits.ac.za (R.S.); neelaveni.padayachee@wits.ac.za (N.P.); 2 Department of Pharmacy Practice, School of Pharmacy, Sefako Makgatho Health Sciences University, Ga-Rankuwa, Pretoria 0208, South Africa

**Keywords:** Central Chronic Medicines Dispensing and Distribution, CCMDD, Dablapmeds, barriers and facilitators, chronic medicine collection, patient medicine parcels, pick-up-points, Implementation science, Consolidated Framework for Implementation Research

## Abstract

South Africa’s (SA) quadruple disease burden comprises communicable diseases such as HIV/AIDS, tuberculosis, maternal and child mortality, non-communicable diseases (NCDs), and injury and violence. Two of these components, HIV and NCDs, generate demand for long-term medicines and are the focus of the review. To improve access to chronic medicines and to alleviate the pressure on overburdened public health facilities, the National Department of Health launched the Central Chronic Medicines Dispensing and Distribution (CCMDD) programme. Stable, compliant patients were granted access to patient medicine parcels (PMPs) via decentralised community pick-up points (PUPs), thereby reducing clinic congestion and improving patient convenience. Despite considerable scale-up, implementation has been inconsistent among provinces. While regional syntheses of differentiated service delivery models exist, no synthesis has yet consolidated the barriers and facilitators specific to the CCMDD across the full decade of its implementation. To understand the barriers and facilitators, a scoping review was conducted for studies published in English between 2014 and 2024. The search was filtered primarily for duplicates, then title and abstract screening, of which 31 articles met the inclusion and exclusion criteria. Data were analysed using inductive thematic analysis; resulting themes were subsequently mapped to the Consolidated Framework for Implementation Research (CFIR) to contextualise findings within implementation science. A grey literature search was also conducted through Google Scholar and Sabinet. Key facilitators of implementation included decentralised service delivery and community-based distribution, reduced patient waiting times, enhanced treatment adherence, reduced patient-borne transport and time costs and improved access to medicines. Limited access to pick-up points in remote areas, miscommunication regarding pick-up dates and poor staff service were cited as barriers. The findings revealed a gap between planned activities and implementation and have significant implications for policy and practice. It further highlighted the need for ongoing optimisation strategies to enhance the programme.

## 1. Introduction

Access to equitable and affordable medicines is a core component towards achieving universal health coverage and the Sustainable Development Goals (SDGs) globally [1]. The World Health Organisation (WHO) estimates that non-communicable diseases (NCDs) account for almost 74% of all global deaths yearly, while sub-Saharan Africa bears over two-thirds of the HIV burden [2]. Management of chronic disease requires long-term engagement with health systems, thereby increasing pressure and overstretching over-burden resource constrained public infrastructure [3]. In response to this challenge, differentiated service delivery (DSD) models became one of the solutions to shift focus to a more client-centred approach while simultaneously reducing the burden on health facilities and reducing barriers to adherence [4].

South Africa (SA) carries a quadruple burden of disease, described as comprising HIV and tuberculosis, maternal and child mortality, non-communicable diseases, and injury and violence. The first and third of these components require sustained pharmacological management and therefore generate the continuous demand for chronic medicines that the CCMDD programme was created to serve [5]. SA has not been an exception to these challenges, with factors such as an increased population size, migration, shortage of healthcare professionals and old infrastructure contributing to longer waiting times for chronic patients accessing care [5]. The healthcare system is characterised by a two-tier public–private structure, with 84% of the population dependent on the tax-funded public sector for care, while the remainder is covered by private medical aid, accessing care predominantly through private providers [6]. Subsequently, SA has made substantial developments to address accessibility to medicine deficiencies and health inequities, and it is within this public health system that the Central Chronic Medicines Dispensing and Distribution (CCMDD) is administered [5,7].

In 2014, through the National Health Insurance (NHI), the CCMDD was developed as a measure to decongest public healthcare facilities and improve access to chronic medicines at designated pick-up points (PUPs) (including ART, antihypertensives, antidiabetics, and other long-term medicines) [8,9]. Phased implementation began initially in 10 NHI pilot districts across eight of the nine provinces, with the Western Cape excluded as it runs its own Chronic Dispensing Unit (CDU). Coverage grew to 16 districts by 2016 and all 46 districts by 2018 [8], reaching over four million patients today [10]. The programme is designed to enable patients to collect their patient medicine parcels (PMPs) from contracted PUPs at specified locations close to their homes or workplace [5,11]. The programme covers the cost of medicine procurement, dispensing and distributing the PMPs and registering new PUPs [10]. Patients have a choice of which PUP they will pick up their parcels from, e.g., facility-based spaced fast lane appointment (SFLA), adherence clubs (ACs), external PUPs (EPUPs) such as community pharmacies, churches, private doctors’ and nurses’ practices, post offices, etc. [8]. By shifting stable patients from facility-based monthly visits to community-based collection, the programme aims to reduce clinic congestion, improve patient adherence through convenience and lower the overall expense of chronic disease management [12]. The programme has grown exponentially since its inception; however, the scale-up has been notably inconsistent between provinces and districts [13].

Similar implementation patterns were identified by Belay and colleagues [4] across sub-Saharan Africa and several of our themes are consistent with their findings. Our contribution focuses on a synthesis of one national programme, across its full ten-year history. This allows us to demonstrate which barriers have endured and which have been resolved over time. The review maps the synthesised themes to the CFIR, indicating the areas where action is most likely to be beneficial. As South Africa moves towards NHI, such evidence is needed to inform policy and service redesign, quality improvement strategies and resource allocation.

This review therefore aimed to identify and map published and grey literature on the barriers and facilitators to CCMDD implementation from the programme’s inception in 2014 to 2024. This review addressed the following questions:

- What are the barriers or facilitators that influence the implementation of the CCMDD programme?
- What are the advancements that have been made to improve the programme since its inception?
- What are the future perspectives of the CCMDD programme’s implementation?

Several of the terms employed in this review are unique to the South African health system. A glossary of key terminology is included at the conclusion of the manuscript for reference.

## 2. Materials and Methods

### 2.1. Step One: Design and Aim

This study used a scoping review methodology, which is the most appropriate design for mapping the nature and extent of evidence for this broad topic. This scoping review mapped evidence on the barriers and facilitators of the CCMDD programme in SA. The five-step methodological framework by Arksey and O’Malley [14], which was further advanced by Levac et al. [15], was used to guide the scoping review. This scoping review was performed in accordance with the Preferred Reporting Items for Systematic Reviews and Meta-Analyses extension for Scoping Reviews (PRISMA-ScR) guidelines [16]. The completed PRISMA-ScR checklist is provided in Supplementary File S1. This review was not registered in a public registry (e.g., PROSPERO, Open Science Framework, Inplasy). As PRISMA-ScR does not mandate registration for scoping reviews and given the exploratory nature of the review objective, formal registration was not carried out. As this scoping review was part of a larger doctoral study, ethical approval was obtained from the University of the Witwatersrand Human Research Ethics Committee (Medical), Certificate No. M240308 M240520-D-0002.

### 2.2. Step Two: Search Strategy

A comprehensive literature search of articles that used qualitative analysis, quantitative analysis, mixed-methods, evaluations, reviews, or policy analysis study designs was conducted in seven electronic databases. These were namely Cochrane Library, EBSCOhost, MEDLINE, PubMed, Science Direct, Scopus, and SpringerLink. A grey literature search was also conducted through Google Scholar and Sabinet to identify reports, theses, and policy documents not indexed in the main databases. The same CCMDD-specific terms were used, and seven records were screened. Two met the inclusion criteria in Table 1. The search included English articles and abstracts published from the period of 2014 to 2024 using a combination of keywords related to chronic medicine dispensing, distribution, barriers, facilitators and CCMDD implementation within the South African context. The final keywords used were “Central Chronic Medicine Dispensing and Distribution”, “CCMDD”, “Dablapmeds”, “pick-up-points”, “adherence club”, “chronic medicine dispensing”, “medicine collection”, “barriers”, facilitators”, “Implementation”, “National Health Insurance pilot” and “South Africa”. These were connected by the Boolean operators (“OR” and “AND”) in a variety of search strings.

The search terms were developed in stages. An initial scoping search was run in PubMed and Google Scholar (specifically for grey literature such as reports, theses and policies) to identify vocabulary used to describe the programme. The CCMDD is a specific national programme with a formal name; therefore, the stated terms were used as primary search anchors. These were combined with general service delivery terms to capture studies that describe the programme without naming it. Broader terms were also tested, including “differentiated service delivery”, “decentralised drug distribution”, “medication refill” and “multi-month dispensing”. Each returned mostly non-South African models that the inclusion and exclusion criteria set out in Table 1 exclude, so they were used only alongside “South Africa” or a CCMDD-specific term.

### 2.3. Step Three: Study Selection

All records retrieved by the first author were exported into EndNote reference manager (version 20), where duplicates were removed. Two independent reviewers screened the titles and abstracts against the inclusion and exclusion criteria. Subsequently, the full text of all potentially eligible articles was retrieved and assessed independently by two authors. A formal inter-rater reliability statistic was not calculated; screening decisions were reconciled by consensus at each stage. Inconsistencies at each step were addressed through dialogue, and a third reviewer was available for adjudication when consensus could not be reached; however, no such cases arose. A PRISMA-ScR flow diagram was used to document and report the screening process.

### 2.4. Step Four: Data Extraction and Charting

Microsoft Excel was used to develop a data extraction tool, piloted on five studies (not included) and refined before full use, to capture the “Barriers to and Facilitators of the Implementation of CCMDD” information. The tool documented the following items: author(s), study title, year, province within South Africa, study design and characteristics, study participants, implementation factors (key barriers/facilitators to CCMDD implementation) and key study findings. Data were separately retrieved by two reviewers and reconciled through conversation. Instances of overlapping data from the same programme phase or cohort were documented to prevent double counting. No conflict arose at this stage of data extraction.

### 2.5. Step Five: Data Synthesis and Analysis

The extracted data were synthesised using a narrative and thematic analysis approach to find common themes and patterns regarding the barriers and facilitators of CCMDD implementation with established guidance [17,18,19]. Barriers and facilitators were coded by inductive thematic analysis, whereby a codebook was repeatedly built from the data rather than from a pre-established framework. The key findings were analysed and categorised into, firstly, subthemes related to each of the main themes (barriers and facilitators), then, secondly, into categories to further break down the concept. As a second step, the analysed themes were matched with the Consolidated Framework for Implementation Research (CFIR) [20,21] to provide a theoretical foundation and facilitate the interpretation of the findings against the CFIR domains in the discussion (Section 4.2). Two reviewers independently screened the titles and abstracts of the studies included from the various databases, with any disagreements resolved in meetings to ensure the accuracy and reliability of theme identification until consensus was reached. This allowed for consistent refinement and grouping of the data into the correct overarching theme. The overall synthesis was reviewed by all members of the research team.

In accordance with PRISMA-ScR guidelines for scoping reviews, a formal critical appraisal or risk-of-bias evaluation of individual sources was not performed, as the objective of this review was to map the extent of available evidence rather than assess its methodological accuracy.

## 3. Results

### 3.1. Study Selection

The initial search across the selected databases yielded a total of 442 potential records. Following the removal of 182 duplicate records, a further 212 were ineligible for the study, as they did not meet the study objective. The remaining 48 records were subsequently screened based on title, abstract and full text. Following full-text screening, 17 articles were further excluded for the following reasons:

- No reference to barriers, facilitators, implementation processes, or system-level outcomes related to CCMDD (*n* = 8).
- Addressed CCMDD implementation within narrow disease-specific contexts without transferable or system-level insights into implementation, which do not allow for generalisations (*n* = 6).
- Study Protocol (*n* = 1).
- Commentaries (*n* = 2).

A final total of 31 studies met the inclusion and exclusion criteria and were included in the scoping review (Figure 1). The remaining 15 references [1,2,3,4,7,8,10,11,12,15,16,17,18,19,20] provide background and methodological context only. The included studies are not numbered consecutively because six of them are also cited earlier for context. In all tables that follow, “Supporting studies” entries refer only to the 31 included studies.

Study Characteristics:

Table 2 presents the characteristics of the 31 included studies. The included studies varied in design, comprising evaluations, reviews, policy analysis, qualitative, quantitative and mixed-methods approaches that provided a multi-dimensional perspective on the operational situations of the CCMDD programme. The included studies were conducted across multiple South African provinces and diverse healthcare settings, allowing for a better understanding of the programme’s performance in both urban and rural contexts.

Studies spanned a decade of CCMDD implementation (2014–2024), with a marked increase in publications from 2020 onwards and a concentration of articles in 2022 (*n* = 12, 38.7%) coinciding with the COVID-19 pandemic period. The majority of studies were conducted in KwaZulu-Natal (*n* = 12), followed by National (*n* = 9), Limpopo (*n* = 2), North West (*n* = 2), Gauteng (*n* = 2) and Mpumalanga (*n* = 1), with the remainder covering multiple provinces or studies at the sub-Saharan level (*n* = 3).

### 3.2. Barriers to CCMDD Implementation

Thematic analysis of the included studies revealed five main barrier domains, each comprising multiple interconnected sub-themes to the effective implementation of the CCMDD programme. A summary of this is shown in Table 3.

#### 3.2.1. Barrier Theme 1: Supply Chain Fragility and Stockouts

Supply chain disruptions emerged as the most frequently documented barrier to CCMDD implementation across the included publications. Bogart et al. [24] described supply chain failures as a significant hindrance to implementation. Stock-outs at PUPs erased patient trust and required patients to return to the facility to collect their medicines, which negated the programme’s primary objective of decongesting clinics. Medication stockouts were cited as a major source of patient frustration by Dorward et al. [26]. This caused impromptu returns to facilities to collect any outstanding medication or buying from private facilities, which defeated the time and cost-saving efforts of the programme.

Muthelo et al. [8] stated that nurses at primary healthcare (PHC) facilities in a district in Limpopo encountered frequent interruptions in dispensing routines due to stock shortages, leading to additional administrative and workload burdens on frontline staff when patients arrived at PUPs and found their medications unavailable. Hlongwana and Gray [12] substantiated these findings at a KwaZulu Natal (KZN) hospital-linked PUP where stock-outs were among the prominent patient-reported barriers to consistent medicine collection.

Inadequate storage facilities at PUPs compounded supply chain challenges in rural Limpopo, with medications sometimes stored under suboptimal conditions that raised concerns about quality and safety [41].

#### 3.2.2. Barrier Theme 2: Health System Capacity and Workforce

Staff shortages, resistance to change, healthcare workers (HCW) workload, inadequate training, poor organisational readiness, infrastructure deficits and inconsistent implementation were frequently cited in multiple studies. One of the most detailed narratives of nurse-level implementation challenges was provided by Muthelo et al. [8]. They described how CCMDD administrative duties were added to existing clinical workloads at facility-based PUPs, previously known as spaced fast-lanes. These duties included preparing PMPs, coordinating with PUP staff and managing returns. No corresponding increase in staffing or allocated time was made.

Unpreparedness for programme demands due to lack of pre-implementation training and insufficient sustained support from programme managers at district level were also reported as barriers. Upon assessing the broader framework of chronic disease management in which CCMDD operates, Lebina et al. [30] discovered that implementation fidelity was consistently low in two South African districts. This was attributed to ineffective training, resource limitations, personnel shortages, and ineffective coordination between district management and facility workers. Leslie et al. [32] evaluated organisational readiness for change implementation in rural PHC facilities in Mpumalanga, discovering significant variability in preparedness ratings among sites. Inadequate leadership support and staff opposition to change were associated with weaker preparedness. Facilities with strong leadership and an engaged workforce showed greater capacity to adapt. Van Heerden et al. [44] identified factors similar to Muthelo et al. as structural barriers that hindered the effective uptake and maintenance of HIV care programmes, directly undermining CCMDD enrolment at the facility level. Sekopa and Netangaheni [41] identified a combination of barriers in Sekhukhune district clinics, including poor physical infrastructure at PUPs, lack of effective referral systems between clinics and PUPs and limited capacity to meet the administrative demands of the programme.

From an implementer viewpoint, Pascoe et al. [13] stated that issues with operational coordination between health facilities and PUPs, including an uneven application of eligibility criteria for CCMDD enrolment, showed systemic weaknesses in programme governance and HCW guidance.

#### 3.2.3. Barrier Theme 3: Patient-Level Barriers

Patient-level barriers constituted a diverse collection of factors inherent to the individual and their social environment. Bogart et al. [24] found that a considerable percentage of eligible patients were unaware of the CCMDD programme or did not fully understand their rights and responsibilities as clients within the programme. Similarly, Hlongwana and Gray [12] recognised poor patient awareness of the CCMDD programme at a hospital in KZN as a barrier to collection.

Barriers related to transportation and geographic accessibility were especially significant in rural and peri-urban areas. Findings from Bassett et al. [11] noted that patients who lived the furthest from external community PUPs had significantly lower uptake in enrolments and collections, emphasising the contradictory issue that community delivery models may be least accessible to patients facing the most significant geographic obstacles. Sekopa and Netangaheni [41] discovered that rural patients in a district in Limpopo encountered exorbitant transport costs and long travel distances to reach PUPs, whilst Mashilo [36] recognised insufficient transportation as a significant issue leading to non-adherence at Tshwane district PUPs.

HIV-related stigma and fear of unintended status disclosure constituted a complex patient-level barrier. While external community PUPs were generally reported to reduce clinic monthly visit stigma, those found in close-knit communities were perceived to increase disclosure risk [25,26]. Comins et al. [25] highlighted this contradiction among female sex workers in KZN, where HIV-related stigma at fixed external PUPs and the pronounced geographic mobility inherent to their profession created significant barriers to CCMDD participation.

Inadequate privacy at certain PUPs and poor communication between patients and providers were cited by Mashilo [36] as additional patient-level barriers, including restrictions to medicine collection, especially for patients who experienced perceived discrimination or judgement from PUP staff.

#### 3.2.4. Barrier Theme 4: COVID-19 and External Disruptions

Five articles identified COVID-19 and pandemic-related disruptions as substantial obstacles to the implementation of CCMDD. The pandemic created a collection of challenges for chronic disease patients who were reliant on decentralised medicine collection. South African lockdown transport-related restrictions severely limited patients’ ability to travel to both clinics and external PUPs, even when services remained technically operational [28,37]. Jarolimova et al. [28] discovered that patients enrolled in the CCMDD programme in KZN experienced significant difficulties accessing PUPs during South Africa’s Level 5 Lockdown due to the near-total halting of public transit and increased police enforcement of movement restrictions.

Fear of contracting COVID-19 at clinic facilities and PUPs was a powerful barrier to accessing services. From the viewpoints of both community members and healthcare workers, Lalla-Edward et al. [29] cited that the fear of infection discouraged patients from collecting chronic medicines at PUPs, resulting in a challenging dilemma between infection prevention and treatment adherence. Boachie et al. [23] quantified the collective effect of these barriers, revealing a notable decrease in chronic services utilisation across multiple chronic delivery models during lockdown. Mboweni and Risenga [37] reported comparable findings in North West province where transport disruptions, financial hardship and fear compounded one another, particularly for patients with multiple chronic conditions requiring regular clinical review.

From a health system viewpoint, HCWs were required to rapidly pivot to differential antiretroviral therapy (ART) administration during the pandemic with insufficient clinical guidance, standard operating procedures or additional staff, as cited by Tlhaku et al. [43]. This adjustment created increased workload pressure and implementation fidelity challenges for an already strained health system. The lack of pre-defined processes for adapting CCMDD during the pandemic was recognised as a structural gap that constrained the programme’s responsiveness to external disruptions.

#### 3.2.5. Barrier Theme 5: Monitoring, Quality Assurance and Clinical Outcomes Discrepancies

A fifth barrier cluster related to discrepancies in clinical outcomes monitoring and quality assurance arose from the review.

Piotie et al. [40] determined that individuals with type 2 diabetes mellitus (T2DM) participating in the CCMDD programme in Gauteng exhibited inadequate glycaemic control as seen by mean glycated haemoglobin (HbA1c) values persisting beyond the recommended threshold despite their enrolment in the programme. This finding indicated that the CCMDD model originally developed for stable HIV-positive patients was insufficiently tailored to meet the intricate monitoring needs of patients with chronic NCDs requiring regular laboratory evaluations, dietary counselling and dose adjustments. Liu et al. [10] reached a similar conclusion in their analysis of the programme’s expansion to non-HIV chronic conditions. They found expansion desirable and feasible from a health system perspective. However, managing co-morbidity and ensuring adequate clinical monitoring in decentralised settings would require substantial adaptation of clinical oversight.

Otwombe et al. [39] noted that a lack of pharmacovigilance systems produced a parallel quality assurance gap where medication errors and adverse drug reactions (ADRs) that occurred in the CCMDD pipeline could not be recognised, examined or used to promote programme improvement. From a provider perspective, Pascoe et al. [13] found that quality of care in decentralised settings was a continuing concern among clinicians who questioned whether patients with complex or evolving health needs were being adequately monitored within the programme’s largely autonomous medicine collection model.

### 3.3. Facilitators to CCMDD Implementation

Five facilitator domains were thematically synthesised with each representing a sub-theme enabling the successful implementation of the CCMDD programme. These are summarised in Table 4 and expanded on below.

#### 3.3.1. Facilitator Theme 1: Patient Convenience and Reduced Treatment Burden

In the included literature, patient convenience and reduced burden of the day-to-day chronic disease treatment cycle were the most consistently reported. The CCMDD programme was intended to enable stable chronic patients to collect medicines at external PUPs closer to their homes or workplaces, at convenient times and without long clinic waits. Studies across diverse populations, settings and methodologies reported that this intention was realised [11,12,13,24,26,34,38]. Reduced transport costs and time saved were among the strongest motivations for patients to remain on the programme. Some participants described collecting their PMPs without taking a full day off work as life-changing [26]. Mokhele et al. [38] quantified patient satisfaction in a multi-provincial survey and found higher satisfaction scores among DSD patients (CCMDD patients included) compared to those accessing care in the clinic-based conventional manner. Reduced waiting times, convenient collection points and respectful PUP staff were commonly cited as reasons for satisfaction.

From a qualitative perspective, Bogart et al. [24] recognised that the programme’s convenience was a compelling facilitator of enrolment among patients who previously struggled to attend clinic appointments due to work constraints, transportation costs or other domestic responsibilities. Hlongwana and Gray [12] found that patients living or working within walking distance of a PUP reported higher satisfaction and more consistent parcel collection. Geographic proximity was therefore a central determinant of programme uptake. Maseko [34] recorded early narratives from patients who described the ability to collect their PMPs from a nearby pharmacy on their way home from work. Patients described the change as transformative: collection was no longer a burden on their time and resources but a routine part of the day. These sentiments were echoed across studies in KZN [24,26], Gauteng [36] and Limpopo [8].

#### 3.3.2. Facilitator Theme 2: Clinic Decongestion and Task-Shifting

Facility decongestion was seen as a health system-level benefit and a great facilitator in multiple publications, demonstrating the strategic rationale that drove CCMDD adoption and its sustained institutional support. By relocating stable chronic patients’ medication collection away from the clinics and into community settings, the programme reduced patient volumes at PHC facilities, thereby freeing up HCW time and space for managing acute illnesses and newly diagnosed patients [8,10,13,38]. Professional nurses at PHC facilities acknowledged that reduced patient volumes on collection days allowed for more time to perform clinical duties despite increased workload challenges that arose from CCMDD administration [8]. District and provincial health managers viewed the programme as a mechanism for improving the efficiency and quality of care provided in overstretched PHC settings through decongestion [13]. Liu et al. [10] further elaborated on how the expansion of CCMDD to include non-HIV chronic conditions amplified the benefit by removing a larger proportion of stable patients, thereby reducing workload. In their scoping review of alternative medicine delivery options, Mash et al. [35] stated that facility decongestion was an ever-present reported facilitator of CCMDD implementation across South African publications, forming a fundamental component of the programme’s health system rationale.

#### 3.3.3. Facilitator Theme 3: Positive Clinical Outcomes

Accumulated data from quantitative and observational research presented a third significant facilitator in the form of demonstrated positive clinical outcomes, lending a hand to the programme’s clinical credibility and policy legitimacy. Bassett et al. [22], in a retrospective study, showed that the kind of ART pickup point, whether clinic-based or community-based, did not substantially affect the 12-month virological suppression rates. The findings were then corroborated by Wang et al. [45] whereby community-based PUP maintained a ≥90% viral suppression rate and had comparable retention to clinic-based standards. In a study on alternative DSD models in KZN, retention on ART and viral suppression were similar to facility-based care [42]. Fox et al. [27] provided the strongest evidence for the efficacy of decentralised dispensing, in the only randomised controlled trial identified in this review. Adherence clubs and fast-tracked dispensing markedly improved patient retention and sustained viral suppression relative to standard care in Gauteng.

Lewis et al. [33] extended clinical evidence by demonstrating that DSD models such as external PUPs produced favourable virologic and retention outcomes not only among first-line ART patients but also among second-line ART regimens, a notoriously complex population. The aggregation of clinical outcome findings from these studies significantly bolstered the programme’s therapeutic validity, advocating for its incorporation into national HIV treatment guidelines and its expansion as a standard model of differentiated care delivery in South Africa.

#### 3.3.4. Facilitator Theme 4: Community Engagement and Support

Community health worker (CHW) involvement in patient tracing, enrolment support and linkage to care was identified as a key enabler by Lebina et al. [30] who found that facilities with stronger CHW integration revealed better implementation commitment in the integrated chronic disease management model. In their sub-Saharan scoping review, Belay et al. [4] reiterated that community was among the consistent facilitators of DSD model implementation encompassing CHW programmes.

Peer support structures such as adherence clubs, which are part of the CCMDD programme, were identified as an enabler of medication adherence, collection and retention. Fox et al. [27] illustrated in their cluster-randomised evaluation that the peer support aspect of adherence clubs, which integrates decentralised medication distribution with organised group support, was independently linked to enhanced treatment engagement, surpassing the advantages of decentralised dispensing alone. Dorward et al. [26] noted a reduction in HIV-related stigma due to external PUP collection situating treatment within a normal community context, thereby reducing clinic-based stigma that had discouraged some patients from attending the clinic consistently. Van Heerden et al. [44] associated community-level interventions, including social mobilisation and patient trust-building activities, as key enablers of HIV care uptake and maintenance at PHC clinics.

For marginalised and special populations, tailored community support proved critical. Comins et al. [25] found that peer-led outreach and mobile pickup alternatives that applied CCMDD principles with stigma vulnerabilities have potential to enhance ART availability for female sex workers in KZN. Community-level adaptability in the DSD model, attuned to local demographic characteristics and epidemiological context, was associated with improved retention and virologic results according to Shigayeva et al. [42].

#### 3.3.5. Facilitator Theme 5: Policy Support and Programme Adaptations

The national policy commitment to the CCMDD programme, along with its proven flexibility to evolving health system demands, represented the fifth facilitator domain. CCMDD was revered as a cornerstone of South Africa’s national strategy to improve the quality and efficiency of medicine use in the public health sector by Meyer et al. [5], establishing an institutional and regulatory structure that conferred legitimacy, resources and a clear implementation mandate. Liu et al. [10] stated that policy enabled scalability through their analysis of its expansion beyond HIV to include hypertension and diabetes mellitus, supported by the National Department of Health that leveraged existing CCMDD infrastructure to further decentralise care.

The programme’s flexibility during the COVID-19 pandemic was a notable example of resilience backed by policy. Tlhaku et al. [43] detailed how HCWs adapted DSD in response to pandemic-era constraints, including the adoption of multi-month dispensing (MMD) to reduce contact frequency. Notwithstanding difficulties with the speed and direction of adaptation, the presence of the CCMDD infrastructure offered a foundation for swift alterations that would not have been accessible without the preceding implementation of the programme. Jarolimova et al. [28] confirmed that CCMDD-enrolled patients fared better in terms of medication continuity during lockdowns than patients in facility-based collection, assigning this advantage to the programme’s established decentralised supply chain.

Flexibility and patient choice, which is a CCMDD design feature enabled by policy, acted as a facilitator of retention, as patients who could transfer to a more conveniently located PUP or clinic were more likely to remain on treatment, as cited by Leslie et al. [31].

## 4. Discussion

### 4.1. Principal Findings

The primary finding of this review is not that the CCMDD is effective, as the clinical evidence has already demonstrated; rather, it is that it is effective in the face of conditions that should be rectifiable. The co-existence of strong clinical data and recorded implementation difficulties indicates that the gap between CCMDD’s potential and its actual impact pertains not to clinic design but to implementation science, health system strengthening, and equity-centred adaptation. A variety of studies confirmed that community-based ART dispensing maintains virological suppression and patient retention equivalent to facility-based care [22,27,33,42,45], yet this success coexists with supply chain failures, health worker strain, patient awareness gaps and organisational unpreparedness throughout the review period, from Meyer et al.’s 2017 analysis [5] to Sekopa and Netangaheni’s 2024 Sekhukhune study [41]. The persistence of these barriers indicates that they are not implementation teething challenges but structural features requiring deliberate intervention. Belay and colleagues [4] found similar patterns across sub-Saharan DSD models, emphasising the fact that the gap between model efficacy and implementation fidelity is a wider regional failure.

The repercussions of unresolved supply chain weaknesses are both severe and specific. Each shortcoming directly undermines the programme’s primary objective of decongestion, as a stockout results in the patient being returned to the facility. This is documented across KZN [12,24,26], Limpopo [8] and Gauteng [36] where procurement planning, demand forecasting and distribution logistics are operationally correctable but have remained insufficiently addressed. Otwombe et al. [39] added an underappreciated dimension; the absence of standardised ADR and medication error tracking at PUPs creates a pharmacovigilance blind spot mismatched with quality standards at facility-based dispensing. Given that CCMDD today includes thousands of pick-up stations catering to millions of patients, a pharmacovigilance infrastructure proportional to this magnitude is urgently required.

Workforce evidence points to the same conclusion from a different angle. Through targeted leadership development and staff motivation strategies, Leslie et al. [32] demonstrated that organisational readiness varies across facilities and is modifiable and measurable. Lebina and co-authors [30] showed that implementation fidelity can be improved through supportive supervision and structured CHW engagement without increasing resources. Muthelo et al. [8] documented the unsustainability of implementation-by-addition, whereby CCMDD administration was absorbed into existing nursing workloads without dedicated time or support. Pascoe et al. [13] further noted provider concerns regarding service quality in decentralised environments, likely reflecting insufficient frontline preparation, highlighting the need for organised, structured training programmes and workload relief as prerequisites. Upon consideration, these results imply a transfer of responsibility from frontline staff to programme design. The issue is that CCMDD was supplemental to clinical work, rather than incorporated into it.

The equity findings carry a parallel consequence for whom the programme currently serves well. Bassett et al. [11] found that patients living furthest from pick-up points, the population the programme was built to reach, had the lowest uptake, which means the current site distribution may be reinforcing the geographic inequity it was designed to reduce. Comins et al. [25] and Mashilo [36] identified access barriers specific to female sex workers and to health literacy constrained patient respectively. Piotie et al. [40] and Liu et al. [10] extend this to treatment quality: patients with NCDs show poorer monitoring and glycaemic control, suggesting that equity in the CCMDD must now be measured in outcomes, not only in access, as the programme expanded beyond HIV.

The COVID-19 evidence base makes the strongest case for converting improvisation into standing policy. Evidence from Jarolimova et al. [28] and Boachie et al. [23] showed that CCMDD patients experienced better medication continuity under lockdown compared to facility-based patients, demonstrating the programme’s value precisely when health systems are under acute strain.

Yet Tlhaku and colleagues [43] show that this resilience was achieved through emergency-adopted multi-month dispensing rather than pre-existing protocol. Transport restrictions, fear of infection, and intensified health worker workloads during the same period [29,37,43] indicate the response was reactive.

Since MMD alleviates patient and systemic burdens without jeopardising virologic results [4,27], the formalisation of MMD as a routine option for all stable CCMDD patients, irrespective of pandemic circumstances, necessitates prompt policy consideration. Although no formal critical appraisal was performed, the evidence varies in strength and should be weighted accordingly. The strongest evidence for the findings on virological suppression and retention is derived from a cluster-randomised trial [27] and four cohort studies [22,33,42,45]. The barrier domains come mainly from qualitative and cross-sectional studies at single sites [8,24,26,36,41,44], which can identify problems but not their national frequency. These barriers should be read as a map of documented challenges, not a measure of prevalence.

### 4.2. Mapping of Themes to CFIR

The ten themes identified through inductive thematic analysis were retrospectively mapped to the five domains of the CFIR [20,21] to provide an implementation science perspective on the findings. This mapping was applied as an interpretive lens following the inductive analysis, consistent with the two-step approach described in Section 2.5 and did not involve re-coding the data.

Barriers were primarily located in the inner setting and outer setting domains. Supply chain fragility and stockouts reflect insufficient available resources in the inner setting, compounded by weak partnerships and connections with external suppliers and contracted service providers in the outer setting. Health system capacity and workforce barriers similarly reflect inner setting resource and cultural constraints, intersecting with the individual domain where gaps in staff capability, motivation and leadership support undermined implementation fidelity. Patient-level barriers, such as transport cost, stigma and low programme awareness, map onto the outer setting construct of patients’ needs and resources, highlighting that many implementation challenges originate beyond the direct control of facilities. The COVID-19 pandemic functioned as a critical external event within the outer setting, exposing the programme’s limited pre-existing guidance for adapting to system-wide disruptions. Weaknesses in monitoring and quality assurance align with the reflecting and evaluating construct of the implementation process domain. They were compounded by an intervention design not originally configured for the more complex monitoring needs of patients with non-communicable diseases.

Facilitators, by contrast, were concentrated within the innovation and outer setting domains. The programme’s adaptability during the COVID-19 pandemic was shaped by the outer setting constructs of the policies and laws and external pressure, which interacted with the innovation domain’s built-in adaptability to enable rapid service modifications.

This mapping indicates that the most persistent obstacles to CCMDD implementation are concentrated in domains that lie outside the direct control of individual facilities. Conversely, the programme’s greatest strengths lie at the innovation-level. This has direct implications for where future improvement efforts are most likely to have meaningful impact.

### 4.3. Strengths and Limitations

The review is strengthened by its comprehensive search strategy, inclusion of multiple methodologies, including grey literature, and its longitudinal perspective across 10 years of implementation experience. Several limitations should be noted. Firstly, publication bias may favour studies reporting negative results. Secondly, the definitions of “barriers” and “facilitators” varied across studies, which constrains direct comparison between them. Thirdly, the evidence base is heavily weighted toward HIV-positive populations, leaving the experiences of patients with non-HIV chronic conditions minimally represented. Fourthly, studies were geographically concentrated in KwaZulu-Natal, which may limit the generalisability of findings to other provinces and implementation in the private sector was minimally captured. Furthermore, the search strategy is itself a limitation. The strategy adopted here was deliberately anchored to CCMDD-specific terminology in order to identify evidence about a specific national programme with a formal name and a widely used brand name (“Dablapmeds”). Studies on similar dispensing models elsewhere, published under different names, may therefore have been missed, along with lessons relevant to the same practice problem. The results should be seen as a description of the CCMDD in particular rather than of decentralised dispensing in general. For this scoping review, no formal protocol was prospectively registered. The absence of formal quality assessment, in accordance with scoping review methodology, necessitates that conclusions be interpreted with an understanding of the differing quality of studies within the included evidence base. An informal indication of which findings rest on stronger and weaker designs is provided at the end of Section 4.1 to assist interpretation.

## 5. Conclusions

This scoping review confirms that South Africa’s CCMDD programme represents a clinically effective, patient-centred and health system strengthened model of decentralised chronic disease care. Across the 31 studies included in the review, the programme was consistently found to maintain virologic suppression and treatment retention while reducing patient treatment burden, clinic congestion and HIV-related stigma. The five key facilitating domains behind the success and further growth of the initiative were identified as patient convenience and reduced treatment burden, clinic decongestion and task-shifting, positive clinical outcomes, community engagement and support and policy support and programme adaptations. However, the review also details a persistent and multi-layered set of implementation challenges that have not been satisfactorily overcome in the programme’s ten-year implementation history. Supply chain fragility and stockouts, health system capacity and workforce, patient-level barriers, COVID-19 and external disruptions and monitoring, quality assurance and clinical outcomes discrepancies constitute five domains of barriers that necessitate focused, sustained, and evidence-based intervention. Addressing these barriers will require domain-specific measures such as the integration of pharmacovigilance and adverse drug reaction reporting directly into the CCMDD workflow as well as tailored clinical monitoring protocols for NCDs. It is evident that standardising PUP staff training and staffing norms may help to build health system capacity.

## 6. Future Directions

Overcoming patient-level obstacles and external shocks such as future pandemics requires documented emergency-adaptation protocols in conjunction with the development of a structured demand-side support strategy, namely community awareness campaigns, transport support, home visits for the vulnerable and stigma-reduction strategies. The CCMDD programme’s potential to serve as a vehicle for universal chronic care, spanning HIV, hypertension, diabetes, and beyond, remains significant within South Africa’s context of high non-communicable disease burden and constrained public health system capacity. Moving forward, the priority is to close the implementation gaps documented in this review before, rather than after, further scale-up. Stronger supply chain governance, standardised workforce capacity and integrated monitoring systems must be built into the CCMDD as NHI expansion proceeds.

## Figures and Tables

**Figure 1:**
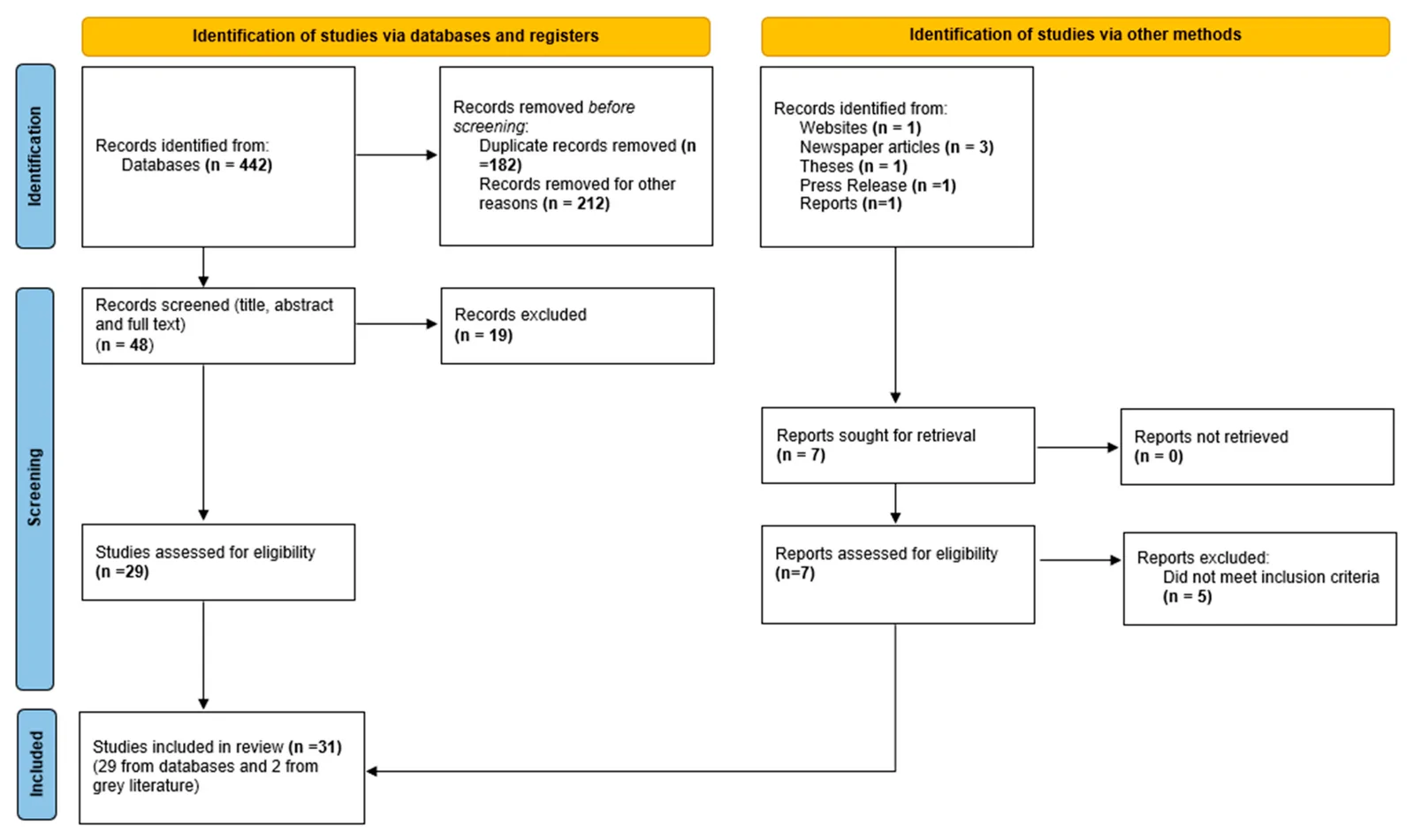
PRISMA-ScR flow diagram.

**Table 1:** Inclusion and exclusion criteria.

Inclusion Criteria	Exclusion Criteria
Articles available in full text and published in English	Were focused exclusively on clinical outcomes without any implementation perspective
Articles published between 2014 (programme inception) and 2024	Non-South African-based articles without direct applicability to CCMDD
Articles that examined barriers, facilitators, implementation processes, or system-level outcomes related to CCMDD	Articles that do not focus on and discuss challenges in medical accessibility.
South African-based articles or focused, in part, on South Africa	Duplicated articles in English
Used qualitative, quantitative, mixed-methods, evaluation, reviews or policy analysis study designs	Conference abstracts, editorials, commentaries, protocols or opinion pieces, without full text
Articles published in peer-reviewed or grey literature	

**Table 2:** Characteristics of the included studies (*n* = 31).

No.	Author (Year)	Location	Study Design	Participants	Key Findings
1	Bassett et al. (2024) [22]	KwaZulu-Natal	Longitudinal observational cohort	ART patients	Community pick-up models’ success. Transportation issues for peri-urban areas.
2	Bassett et al. (2022) [11]	KwaZulu-Natal	Cross-sectional survey	ART patients	Site-level differences and communication gaps impacted patient outcomes. Viral suppression was unaffected by PUP type.
3	Belay et al. (2022) [4]	Sub-Saharan Africa	Scoping review	Policymakers; providers	Variable PUP quality. Health system capacity gaps.
4	Boachie et al. (2023) [23]	National	Retrospective analysis	Aggregated data on CCMDD-enrolled chronic patients	COVID-19 lockdown led to missed collections and reduced access to CCMDD services.
5	Bogart et al. (2022) [24]	KwaZulu-Natal	Qualitative implementation evaluation	Patients; providers; programme managers	Barriers reduced programme sustainability. Stakeholder engagement; digital integration.
6	Comins et al. (2022) [25]	KwaZulu-Natal	In-depth Interviews	Female sex workers; clinicians	Community-tailored approaches improved access to vulnerable groups.
7	Dorward et al. (2020) [26]	KwaZulu-Natal	In-depth interviews and focus group discussions	Patients; nurses; pharmacists	Reduced clinic congestion due to community-based PUPs.
8	Fox et al. (2019) [27]	National	Cluster randomised evaluation	ART patients; clinicians	Improved retention due to task shifting in adherence clubs.
9	Hlongwana & Gray (2022) [12]	KwaZulu-Natal	Observational cross-sectional comparative study	Pharmacists; nurses; patients	Ease of access for patients at PUPs facilitated medicine collection.
10	Jarolimova et al. (2022) [28]	KwaZulu-Natal	Interviews	CCMDD patients and providers	COVID-19 lockdown disruptions resulted in uncertainty and hindered access to treatment for patients.
11	Lalla-Edward et al. (2022) [29]	Gauteng & Mpumalanga	Cross-sectional exploratory	Community members; healthcare workers	COVID-19 interruptions affected both staff and patients.
12	Lebina et al. (2019) [30]	Gauteng & North West	Cross-sectional study	Facility managers; nurses; pharmacists	Inconsistent implementation fidelity. Differing levels of facility assistance and resource availability.
13	Leslie et al. (2022) [31]	North West	Retrospective secondary analysis	ART patients	Flexibility in choice of service at CCMDD sites.
14	Leslie et al. (2022) [32]	Mpumalanga	Quantitative assessment	PHC facility managers; staff	Organisational readiness in rural areas.
15	Lewis et al. (2021) [33]	KwaZulu-Natal	Retrospective cohort study	PLHIV receiving second-line ART	Differentiated care contributes to viral suppression and patient retention.
16	Liu et al. (2021) [10]	National	Descriptive analysis	Policymakers; implementers	Expansion of CCMDD programme to include other chronic conditions key to management of comorbidity.
17	Maseko (2018) [34]	National	Programme report	Policymakers; patients	Reduced treatment burden and patient satisfaction.
18	Mash et al. (2021) [35]	National	Scoping review	Healthcare providers; policymakers	CCMDD as the primary alternative medicine delivery model.
19	Mashilo (2024) [36]	Gauteng	Descriptive non-experimental survey	Chronic patients	Low health literacy contributed to non-adherence at CCMDD pickup points.
20	Mboweni & Risenga (2023) [37]	North West	Qualitative phenomenological	Chronic disease patients	COVID-19 lockdown revealed CCMDD’s vulnerability to external shocks.
21	Meyer et al. (2017) [5]	National	Principally a structured review	Pharmacists; policymakers	Improved medicine distribution due to supportive policy frameworks.
22	Mokhele et al. (2024) [38]	National	Quantitative survey with qualitative content analysis	ART clients	Higher satisfaction among CCMDD clients vs. clinic-based patients.
23	Muthelo et al. (2020) [8]	Limpopo	Qualitative descriptive, phenomenological and exploratory design	Professional nurses	Workforce dissatisfaction and insufficient training.
24	Otwombe et al. (2022) [39]	National	Descriptive cross-sectional survey	Pharmacists; programme managers	Underreporting of adverse drug reactions and medication errors in CCMDD programme.
25	Pascoe et al. (2020) [13]	National	Cluster-randomised evaluation	Patients; providers; implementers	Heterogeneity in the application of eligibility criteria.
26	Piotie et al. (2021) [40]	Gauteng	Retrospective record review	Diabetes patients	Suboptimal clinical management; programme not adapted for monitoring complex non-HIV conditions.
27	Sekopa & Netangaheni (2024) [41]	Limpopo	Qualitative	Nurses; CCMDD Clients	Human resource and logistical constraints are major obstacles to CCMDD implementation.
28	Shigayeva et al. (2022) [42]	KwaZulu-Natal	Retrospective cohort	ART patients	Strong retention and viral suppression rates in CCMDD.
29	Tlhaku et al. (2024) [43]	KwaZulu-Natal	Qualitative	Healthcare workers	COVID-19 forced operational adaptations.
30	Van Heerden et al. (2022) [44]	KwaZulu-Natal	In-depth interviews	Clinic managers; patients	Limited staff capacity and facility environment affected uptake and maintenance.
31	Wang et al. (2023) [45]	KwaZulu-Natal	Secondary analysis of trial cohort data	ART patients	Improved retention in care and viral suppression.

**Table 3:** Main barriers to CCMDD implementation.

Barrier Theme	Sub-Themes	CFIR Domain (s)	Supporting Studies [Refs.]
Supply chain fragility and stockouts	Delays in patient medicine parcel delivery. Inconsistent medicine availability. Inadequate storage at pickup sites. Supplier delays. Medicine stock-outs at service provider.	Inner Setting (primary); Outer Setting (secondary)	[8,12,24,26,35,36,39,41] ^a^
2. Health system capacity and workforce	Staff shortages. Resistance to change. Quality variability at PUPs. Lack of policy enforcement. Inconsistent implementation across sites. Inadequate training in CCMDD procedures. Insufficient infrastructure. Weak inter-facility communication and referral systems.	Inner Setting: Individuals	[5,8,10,13,30,31,32,38,41,44]
3. Patient-level barriers	Limited awareness and knowledge of CCMDD. Missed appointments. Transportation and distance challenges. Stigma and fear of unintended status disclosure. Fragmented care for patients with comorbidities.	Outer Setting	[4,11,12,13,22,24,25,34,35,36,37,41]
4. COVID-19 and external disruptions	Fear of infection at health facilities. Lockdown-related transport restrictions. Rapid adaptation without adequate guidance.	Outer Setting	[23,28,29,37,43]
5. Monitoring, quality assurance and clinical outcomes discrepancies	Insufficient pharmacovigilance systems. Underreporting of adverse drug reactions and medication errors. Suboptimal glycaemic control in chronic disease patients. Inadequate clinical monitoring for co-morbid patients.	Implementation Process; Innovation	[10,13,27,33,39,40,42,45]

^a^ reference numbers denote all studies contributing evidence to the overall theme; citations for individual illustrative sub-themes are given in the narrative text below the table.

**Table 4:** Main facilitators to CCMDD implementation.

Facilitator Theme	Sub-Themes	CFIR Domain (s)	Supporting Studies [Refs.]
Patient convenience and reduced treatment burden	Proximity of PUPs to home and work. Reduced clinic waiting times. Cost savings on transport. Time saved. Flexible and longer collection times. Reduced need for leave to attend clinics.	Innovation; Outer Setting	[11,12,13,24,26,34,35,36,38] ^b^
2. Clinic decongestion and task-shifting	Reduced clinic congestion. Freed time for HCWs for clinical consultations. Improved staff capacity to manage acute illnesses. Task-shifting of stable chronic patients to the community level.	Inner Setting; Implementation Process	[8,10,13,35,38]
3. Positive clinical outcomes	Equivalent virological suppression to clinic-based care. Retention on HIV treatment. Non-inferior clinical outcomes in second-line ART patients. Clinical effectiveness in a variety of geographic areas.	Innovation	[22,27,33,42,45]
4. Community engagement and support	Community health worker involvement. Adherence clubs and peer support groups. External PUPs reducing clinic-associated stigma. Non-governmental organisation partnerships. Patient empowerment.	Outer Setting; Implementation Process	[4,25,26,27,30,42,44]
5. Policy Support and programme adaptations	National Department of Health backing. Programme scalability to include NCDs. Existing infrastructure enabling COVID-19 adaptation. Multi-month dispensing capacity. Integration with PHC system.	Outer Setting; Innovation	[5,10,31,32,43]

^b^ reference numbers denote all studies contributing evidence to the overall theme; citations for individual illustrative sub-themes are given in the narrative text below the table.

## Data Availability

No new data were created or analyzed in this study. Data sharing is not applicable to this article.

## Supplementary Materials

The following supporting information can be downloaded at https://www.mdpi.com/article/10.3390/pharmacy14070143/s1. Supplementary File S1: PRISMA-ScR Checklist.

## Glossary of Key Terminology

The following terms and abbreviations are used in this manuscript:

**Abbreviation/Term**	**Definition**
Central Chronic Medicines Dispensing and Distribution (CCMDD)	South African national programme for the centralised dispensing and decentralised distribution of chronic medicines, branded “Dablapmeds”.
Chronic Dispensing Unit (CDU)	The equivalent programme operated independently by the Western Cape province.
Consolidated Framework for Implementation Research (CFIR)	A widely used implementation science framework that helps researchers and practitioners explain and predict barriers and facilitators to the success of an intervention.
Community Health Worker (CHW)	Frontline health worker who is a trusted member of the community and has a good understanding of the communities they serve.
Differentiated Service Delivery (DSD)	An approach that adapts HIV services to patients’ needs and preferences while easing the burden on the health system.
Multi-Month Dispensing (MMD)	Allows stable patients to collect a three- to six-month supply of chronic medication.
National Health Insurance (NHI)	South Africa’s strategy to achieve universal health coverage.
Patient Medicine Parcel (PMP)	Pre-packed patient medication delivered to convenient PUPs for stable chronic patients.
Preferred Reporting Items for Systematic Reviews and Meta-Analyses extension for Scoping Reviews (PRISMA-ScR)	Checklist designed to improve the transparency, completeness, and consistency of reporting in scoping reviews.
Pick-Up Point (PUP)	Any contracted site, facility-based or external, at which a patient collects a patient medicine parcel.
Spaced Fast Lane Appointments (SFLA)	A facility-based PUP collection arrangement without a clinical consultation.
